# Validation of a novel NGS based *BCR::ABL1* kinase domain mutation detection assay in Indian cohort

**DOI:** 10.1038/s41598-024-66310-8

**Published:** 2024-07-08

**Authors:** Pooja Chaudhary, Spandan Chaudhary, Falguni Patel, Shiv Patel, Toral Vaishnani, Nikha Trivedi, Dhiren Patel, Tushar Sonagara, Ashish Hirapara, Kavisha Vyas, Lokesh Patel, Raja Kumar, Nikkan Chakraborty, Divya Sharma, Jigar Suthar, Payal Kamdar, Ekta Jajodia, Firoz Ahmad, Neeraj Arora

**Affiliations:** 1 Molecular Department, Unipath Specialty Laboratory Ltd, Ahmedabad, Gujarat India; 2 Department of Biotechnology and Microbiology, Shri M.M. Patel Institute of Science and Research, Kadi Sarva Vishwavidyalaya, Gandhinagar, Gujarat India

**Keywords:** Cancer genomics, Molecular biology

## Abstract

The efficacy and treatment outcome of a CML patient are heavily dependent on *BCR::ABL1* kinase domain (KD) mutation status. Next-generation sequencing technology is a bright alternative to the previously used sanger sequencing method due to its global presence in diagnostic setups, massive parallel sequencing ability, and far better sensitivity. In the present study, we have demonstrated a new protocol for kinase domain mutation analysis using the next-generation sequencing (NGS) method using the ion torrent sequencing platform. This protocol uses RNA as the starting material, followed by nested PCR to amplify the fusion transcript, which is subsequently used as a template for NGS. Initial validation and comparison of this assay with the sanger sequencing (SS) method yielded 95.23% agreement. CML samples (n = 121) with a failure to TKI response were subjected to this newly developed NGS-based assay to detect KD mutations, from which samples were found to have mutations with a sensitivity ranging from 2.32 to 93.41%. A total of 34.71% of samples (n = 42) were found to be positive for one or more KD mutations, whereas 65.29% of samples (n = 81) were found to be negative. Nine samples out of 42 positive samples, i.e., 21.42%, were found to have compound mutations. This is one of the first studies from India, which includes more than 160 samples and is analyzed by the NGS approach for KD mutation analysis.

## Introduction

Chronic myelogenous leukemia (also called CML or chronic granulocytic leukemia) is a myeloproliferative disorder and most common blood cancer characterized by the presence of translocation t(9;22) (q34;q11), which generates the Philadelphia (Ph) chromosome and the associated fusion gene *BCR::ABL1*. Tyrosine kinase inhibitors (TKIs) are a type of targeted therapy to treat CML. TKIs target the abnormal BCR::ABL1 protein that causes uncontrolled CML cell growth and block its function, which eventually leads to cell death. Imatinib (Glivec; Novartis), the first tyrosine kinase inhibitor (TKI), approved in 2001 in Europe and the United States, has completely changed patients’ life expectancy^1^. This drug is recommended as the first line of therapy for all CML phases and, as its patent has expired, is now available as a generic drug. For the patient resistant to Imatinib and other first-line treatments, second-line treatment was approved as two second-generation TKIs, Dasatinib (Sprycel; Bristol-Myers Squibb) and Nilotinib (Tasigna; Novartis), in the United States and Europe between 2006 and 2007. Dasatinib was approved for CML patients with all disease phases, and Nilotinib was only approved in the chronic phase (CP) and accelerated phase (AP). For all the CML adult patients with CP, AP, or blast phase (BP) Ph + CML who are resistant to, or intolerant of, first and second generation TKIs, another second-generation TKI, Bosutinib (Bosulif; Pfizer), was licensed in the United States in 2012 and in Europe in 2013^2,3^. For all the CML adult patients with CP, AP, or BP in Ph + CML who are resistant to second-generation TKIs, the third-generation TKI, Ponatinib (Iclusig; ARIAD), was approved in the United States in 2012 and in Europe in 2013. These three generations of tyrosine kinase inhibitors had dramatically changed the management and long-term survival of patients affected by chronic myeloid leukemia (CML)^4^. Along with survival, resistance has also been observed^5–8^.

In approximately 33% of patients who experience resistance to first-line therapy and in up to 50% of patients who experience resistance to second- or subsequent-line therapy, point mutations in the *ABL1* kinase domain (KD) that impair TKI binding can be detected^9^. The resistance rates may be underestimated because of the limited sensitivity of SS method^10^, as low-level mutations cannot be identified. Mutations may arise at critical contact points between the inhibitor and its target or in key regions of the KD, namely the phosphate-binding loop (P-loop), the catalytic cleft, or the activation loop (A-loop)^9^. There are various mechanisms that lead to a decrease or loss of response to TKIs, but the acquisition of point mutations in the *BCR::ABL1* kinase domain (KD) is the most important and probably the only actionable one^10^. Studies have already established a spectrum of sensitive and resistant mutations and mutants to Imatinib and second- and third-generation TKIs^11^. Mutations make the drug ineffective in obtaining a deep clearance of cells with *BCR:ABL1* fusion, which slows down the clinical response and also accelerates the acquisition of additional mutations^12–14^. The resulting effect can be a clonal complexity in some patients, which is a difficult phenomenon to address therapeutically^15^. This is the reason that the European Leukemia Net (ELN)^16^ and the National Comprehensive Cancer Network^17^ both have recommended screening for mutations in case of failure and warning of response to the drug under treatment.

Several assays have been designed and validated for KD-resistant mutation detection in patients with CML, but SS is the current gold standard method. SS is a faster and more cost-effective method, but due to its low sensitivity with a mutation detection limit of 10 to 20%^18–26^, it falls behind in detecting low-level mutations. The SS method provides only rough estimates of mutated clone abundance, and it is also a fact that it cannot differentiate between polyclonal and compound mutations unless it is preceded by a step of cloning, which is not a routine practice in the majority of diagnostic labs. Some studies have reported a more sensitive mass spectrometry assay for KD mutation detection with a lower detection limit of 0.2% for the majority of mutations, but its availability was a limiting factor^26^. Since the emergence of next-generation sequencing (NGS) technology and its proven advantages like depth and massive parallel approach, it has been well received in the routine diagnostic workflows in the hematology and oncology segments. Sequencing multiple fragments together at a significant depth makes the NGS a very suitable method to detect even multiple mutations with greater sensitivity^23^. High sensitivity gives the NGS method an edge in picking up emerging mutations a few months earlier than other methods^20,22^. Low-level (1%) mutations that make patients resistant to TKIs are routinely picked up by the NGS method, which is not possible to detect by the SS method. The aim of this study was to develop an NGS-based kinase domain mutation detection assay that can detect mutations at a low level in patients, which are usually left by the traditional SS method. There are several similar assays that have been developed using various sequencing platforms like Roche, Illumina, and Ion Torrent. Protocols developed on Roche^27^ and Illumina^28^ platforms have used RNA as a starting material and a protocol on Ion torrent platform^28^ uses DNA as starting material. As per our review of the literature, no assay is available that uses RNA and is on the Ion Torrent platform. The advantage of this assay is that it does not need to be run individually; instead, it can be accumulated with any other Thermofisher panel-based assay that uses 500 flows for sequencing. In this study, we developed and assayed as per the recommendation for NGS KD mutation testing in CML patient^4^ on the Ion Torrent sequencing platform, which has wide accessibility in diagnostic settings globally.

## Methods

### Sample details

**Figure 1:**
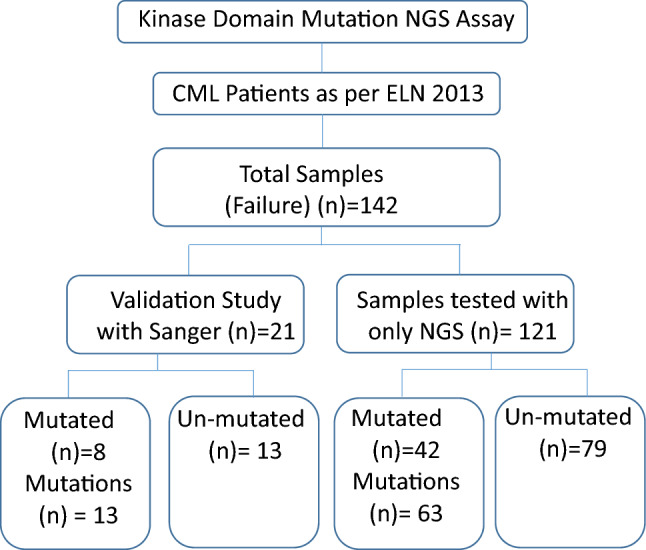
Study flow chart, overview of the design and distribution of the patients and samples analyzed by our nested PCR and NGS-based methods. ELN: European Leukemia Network; NGS: Next Generation Sequencing.

This assay is designed at Unipath Specialty Laboratory Ltd., which routinely performs kinase domain mutation analysis by the SS method. For the initial standardization and validation of the assay, we selected 21 left-over samples from the samples registered for kinase domain mutation testing by the SS method. After the assay was validated, more than 121 samples were subjected to mutation detection using an in-house developed kinase domain mutation detection assay. All methods were performed in accordance with the declaration of Helsinki. This is a retrospective study that used left-over samples, and patient details are anonymized. A detailed study flow chart is presented in Fig. 1. This study was approved by the Sangini Hospital Ethics Committee (ERC/147/Inst/GJ/2013/RR-19). The ethical committee has waived off the requirement of the patient consent forms for this study, due to its retrospective design and use of anonymized patient data, in compliance with ethical principles and regulations. Strict protocols have been put in place to guarantee the privacy and confidentiality of all patient data during the course of the study, however.

### SS based kinase domain mutation detection analysis

Sanger sequencing of *BCR::ABL1* kinase domain was performed using ABI 3500 sequencer as per the protocol described^29^.

### Primer designing

**Figure 2:**
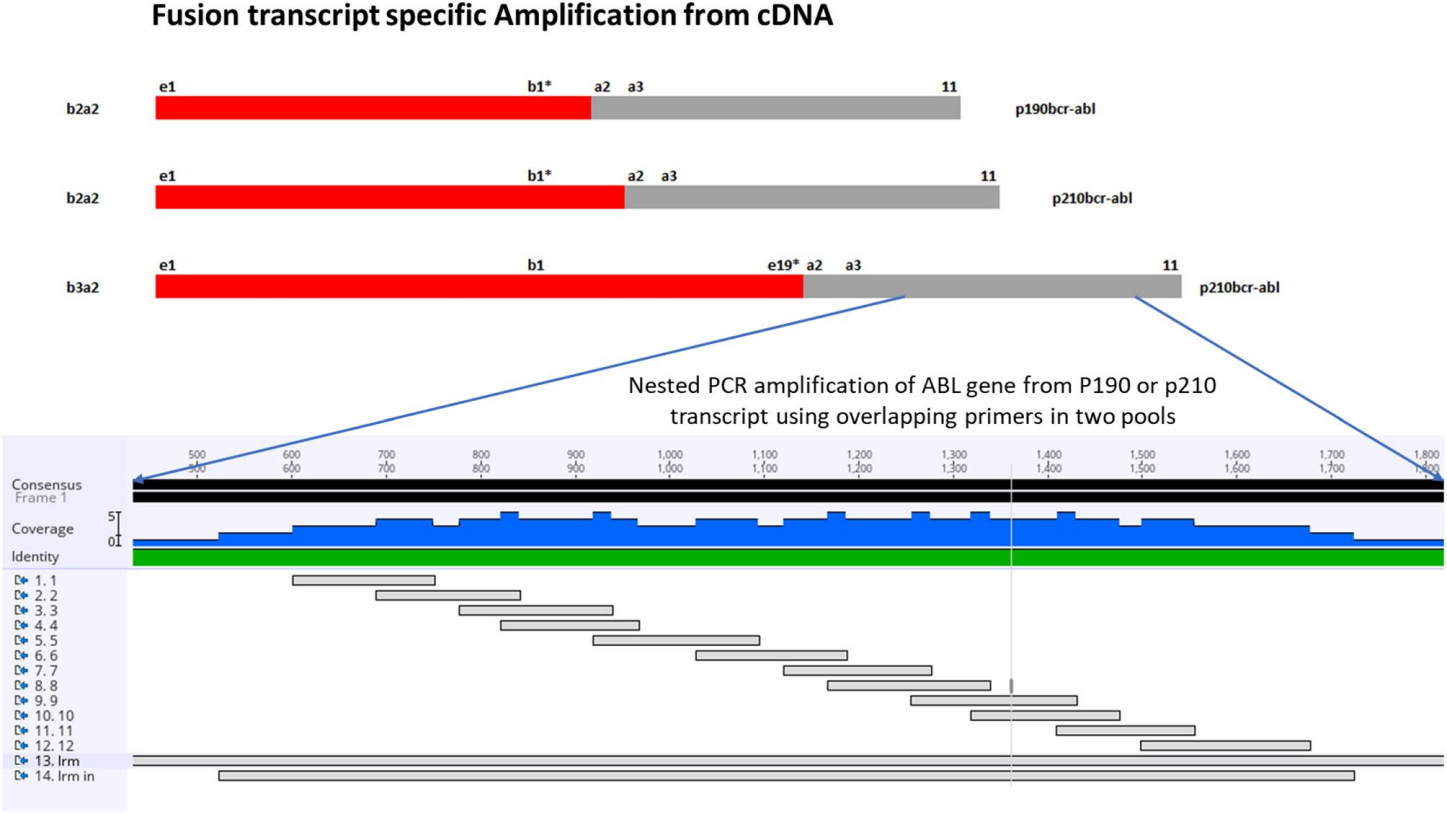
Schematic representation of the structure of the fusion transcripts P190 and P210, along with the mapping of primer pairs on the *ABL* gene region.

This assay uses primers at two points: first for the specific amplification of the transcript in question (P190 or P210), which uses the primers from a published study^29^ and second for the amplification of the *ABL* region of fusion. Primers for the amplification of the *ABL* region were designed in-house to cover the entire KD region of the *ABL* gene by using the transcript NM_005157.6 as a reference. These primers were designed to capture the region from codon 160 to 500, which is approximately 1020 bp of the transcript. A total of 12 primer pairs were designed in an overlapping manner to ensure no gap in the entire region. A schematic diagram of the primer design and covered region of the mentioned transcript is provided in Fig. 2. Primer sequences can be made available upon request.

### NGS based BCR-ABL Kinase domain mutation analysis

#### Library preparation and sequencing

**Figure 3:**
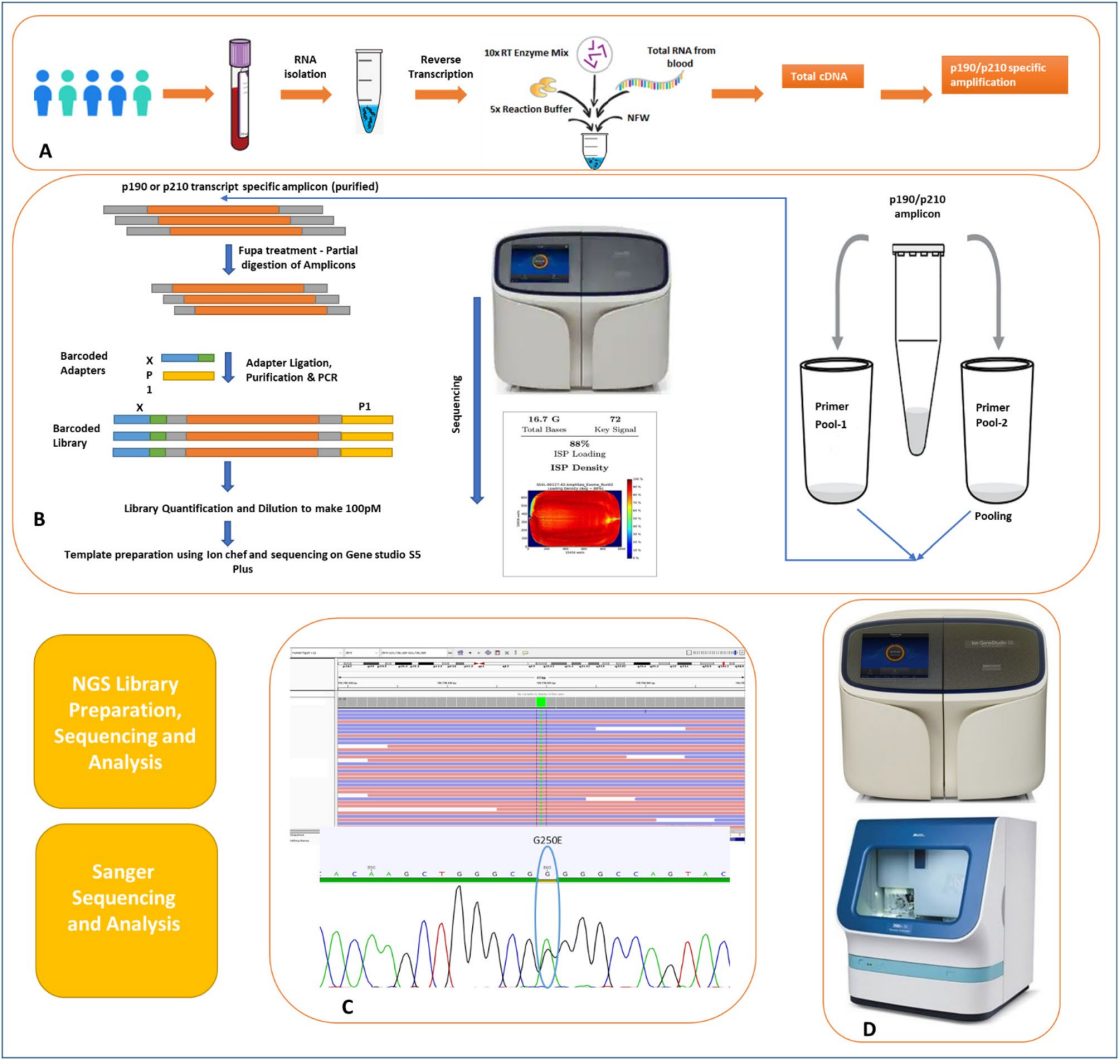
Validation of the developed NGS assay with Sanger sequencing confirmed CML samples. **A** Process flow from sample collection to transcript-specific amplicon generation, including RNA isolation, reverse transcription reactions to generate cDNA, and subsequent transcript-specific amplification. **B** Amplicons generated were amplified in two separate reactions by two primer pools, which will be pooled together for further library preparation as per the protocol of the Ion AmpliSeq™ Library Kit. Prepared libraries were sequenced using the Ion Torrent Gene Studio S5 Plus platform. **C** Representative image of comparison view of same variant detected by sanger sequencing and IGV view of ngs data **D** Sanger sequencer and Ion Torrent Gene Studio S5 Plus sequencer.

For NGS-based *BCR::ABL1* kinase domain mutation analysis, blood collected in an EDTA or paxgene tube was used as the starting material. RNA was extracted using the QIAamp RNA Blood Mini Kit (Cat no. 52304), followed by cDNA preparation using the NGS Reverse Transcription Kit (Cat no. A45003). 500 ng to 1 µg of total RNA was used to prepare cDNA. The cDNA was then subjected to amplification using fusion-specific primers, and the resulting amplicon was subjected to library preparation using the Ion AmpliSeq™ Library Kit Plus (Cat. No. 4488990) as per the manufacturer's instructions. *BCR::ABL1* kinase domain mutation assay-specific primers are divided into two pools to restrict unwanted amplification of adjacent regions. A detailed protocol is provided in the supplementary material, and a schematic diagram of the process flow is provided in Fig. 3. Libraries prepared were then subjected to quantification using the Ion Library TaqMan™ Quantitation Kit (Cat. no. 4468802), followed by pooling, bead-based clonal amplification, and chip loading using the Ion Chef instrument (Cat. no. 4484177). All the libraries were targeted to generate a minimum of 0.5 million reads. The generated data was then analyzed using ion reporter software. Amplicons used in this assay for amplifying KD regions have a size range of 200–250 bp, which allows pooling of this assay with any other themofisher panels that use 500 sequencing flows. This gives an added advantage to any lab that routinely uses themofisher panels for sequencing on Ion Torrent.

### Data analysis

A minimum of 0.1 million reads was considered for all the samples; samples with fewer than 0.1 million reads were subjected to repeat sequencing. Percent reads mapped on target achieved was > 99% for all the samples; the average base coverage depth was around 1900 to 2000x. All the reported variants were verified by integrated genome viewer (IGV) analysis. The sequencing coverage of the *ABL* gene was confirmed by torrent suit software, and it was observed that the entire targeted region of the *ABL* gene was captured without any gaps. For all the samples, analysis was performed manually to check the hotspot mutations analyzed by the IonReporter™ (IR) Software 5.18.2.0 using bed files targeting the desired region of the *ABL* gene as a reference. This file was also mapped for all 110 kinase domain hotspot mutations reported so far^4^. All hotspot mutations analysed in the assay are provided in Table 1.

**Table 1:** List of mutations/variants covered by the newly developed NGS assay (This table is adopted from a previously published study)^4^.

Mutations poorly sensitive to Imatinib	M237V, I242T, M244 V, K247R, L248V, G250E, G250R, Q252R, Q252H, Y253F, Y253H, E255K, E255V, E258D, W261L, L273M, E275K, E275Q, D276G, T277A, E279K, V280A, V289I, V289I, V289A, E292Q, E292V, I293V, L298V, F311L, F311I, T315I, F317L, F317V, F317I, F317C, Y320C, L324Q, Y342H, M343T, A344V, A350V, M351T, E355D, E355G, E355A, F359V, F359I, F359C, F359L, D363Y, L364I, A365V, A366G, L370P, V371A, E373K, V379I, A380T, F382L, L384M, L387F, L387V, M388L, H396R, H396P, H396A, A397P, S417F, S417Y, I418S, I418V, A433T, S438C, E450K, E450G, E450A, E450V, E453G, E453A, E453K, E453V, E453Q, E459K, E459V, E459G, E459Q, M472I, P480L, F486S
Mutations poorly sensitive to Dasatinib	V299L, T315I, T315A, F317L, F317V, F317I, F317C
Mutations poorly sensitive to Nilotinib	Y253H, E255K, E255V, T315I, F359V, F359I, F359C
Mutations poorly sensitive to Bosutinib	E255V, E255K, V299L, T315I
Mutations poorly sensitive to Ponatinib	T315M, T315L

Around 0.5 million reads were generated, covering the entire region at an average depth of 2000X. The sequencing reads, QC, mapping of the hg19 human reference genome, variant calling, and annotation were carried out with IonReporterTM (IR) Software 5.18.2.0. Latter uses different databases for the identification and characterization of gene-associated variants. The annotation for variants was derived using various disease databases like ClinVar. The population frequency information from 1000 genomes (ExAC, GnomAD, and ESP) was used for the elimination of common variants and polymorphisms. For the prediction of the possible impact of coding non-synonymous SNVs on the structure and function of a protein, PolyPhen-2 and SIFT scores were used. Further Oncomine Reporter software was used for annotating variants with a curated list of relevant labels, guidelines, and global clinical trials.

### Assay validation: accuracy, sensitivity and reproducibility

#### Validation experiment

The current study was validated with 21 known samples of CML, including 13 known negative and 8 known positive samples of CML patients. In these known samples, KD mutations were characterized using the SS method. A total of eight positive samples used for KD mutations were reported to have failed or were in the warning stage of CML disease. A representative chromatogram profile of the sanger data and the IGV of identified mutations for the validation samples are represented in Fig. 3.

#### Sensitivity determination experiment

**Figure 4:**
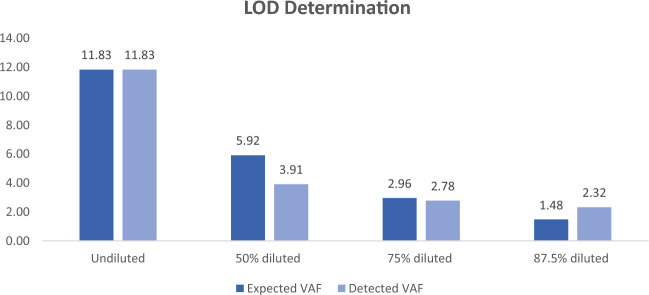
Results of the experiments for determining the limit of detection. The graph represents the detected and expected variant allele frequencies (VAF) of the CML positive sample, which was serially diluted three times in a 1:1 ratio with the negative sample.

For sensitivity determination, one positive sample was selected with variant F317L at 11.83% VAF, which was serially diluted in a 1:1 ratio with the RNA sample of a known negative patient for further three times (supplementary Table 1). Diluted samples were subjected to cDNA preparation, transcript enrichment, library preparation, data generation, and analysis as mentioned in the methodology segment. This means the variant should also be diluted from 11.83 to 5.92%, 2.96, and 1.48%, respectively, in three consecutive 1:1 dilution, but instead the assay detected variants at 3.91, 2.78, and 2.32% (Fig. 4). The reason for the difference between expected and detected VAF can be the variation in the number of reads generated, but variant detection was correct. So we determined the limit of detection for the assay as 2%, below which variants detected will not be considered. It is worth noting that the majority of studies have defined the NGS-based KD mutation assay sensitivity as 1–3%^4^.

#### Reproducibility experiment

**Figure 5:**
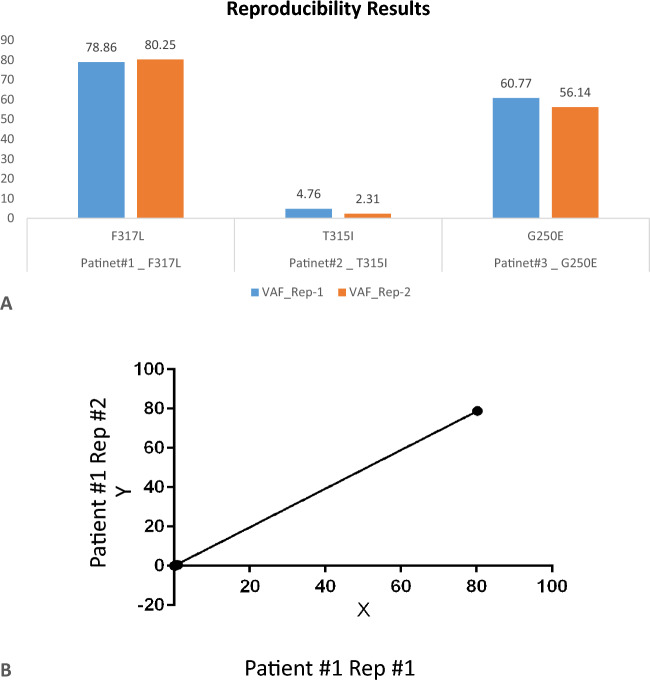
**A** Graphical representation of the experiment conducted for reproducibility of the assay. This graph represents results for only positive samples with the VAF detected in two individual runs with different libraries. **B** Regression analysis performed on one representative positive sample with all the polymorphisms and respective allele frequencies showed significant results.

A total of 5 patient samples were used for reproducibility experiments, which included three positive and two negative samples. All the samples were processed as total RNA for data generation as per the methodology mentioned and subsequent analysis. Three positive samples selected had allele frequencies of 78.86, 4.17, and 60.77%, whereas in the repeat experiments, results were obtained of 80.25, 2.31, and 56.14%, respectively, for the same three samples (Fig. 5A). Variation in the VAF was detected at 0.69, 1.22, and 2.31%, which were within acceptable limits. All negative samples were detected as negative in repeat experiments. This means that the assay has good reproducibility with 100% concordance in overall results. For one positive sample, regression analysis was also performed with all the variants derived from two replicates. The graph of the same is presented in Fig. 5B, and it had a slope of 0.9828 ± 0.001, which is significant, and an R2 value of 0.99 for goodness of fit analysis, which concludes good reproducibility. The data from the reproducibility experiments is presented in supplementary data (Table 2).

**Table 2:** Newly developed NGS based assay specifications in comparison with SS.

Criteria	True positive (a) = 7	False positive (b) = 1
	False negative (c ) = 0	True negative (d) = 13
Sensitivity	[a/(a + c)] × 100	100%
Specificity	[d/(b + d)] × 100	92.86%
PPV	[a/(a + b)] × 100	88%
NPV	[d/(c + d)] × 100	100%

## Results

### Sanger validation results

21 known clinical samples (SS confirmed) considered for validation using KD mutation analysis by the NGS method resulted in 95.23% concordance. A total of 8 samples were known positive, while the remaining 13 were known negative. In the validation study, NGS and SS results were found to be concordant for all positive samples and 12 negative samples, except one. One sample, which was negative for SS, was found to have an L298R mutation with 3.85% VAF, which is less than the limit of detection for SS. In two out of eight samples found positive by SS, additional mutations were detected by the NGS method, which were at a lower frequency than the limit of detection of SS and were at < 15–20% VAF (supplementary data, Table 3). If sanger is considered the gold standard method by assuming that one sample is falsely positive by the NGS method, then the sensitivity and specificity of this assay were determined to be 100 and 92.86%, respectively, whereas the positive predictive value (PPV) and negative predictive value (NPV) of the NGS-based assay would be 88 and 100%, respectively (refer to Table 2). However, if we consider the outlier sample as true positive, the VAF of the detected variant is less than the limit of detection of the SS method then, all four criteria (sensitivity, specificity, PPV, and NPV) of the NGS assay will be 100%. The assay sensitivity, specificity, PPV, and NPV were calculated as described earlier^30^.

**Table 3:** Features of the patients included in the present study.

Features	Samples (n)
Total samples	121
Female	40 (33.06%)
Male	81 (66.94%)
Positive	42 (34.71%)
Negative	79 (65.29%)
Median age	40 (14–72)
Disease phase/type	
CML	121
Failure	121
Warning	0

### NGS assay results

Out of 121 samples included in the study, 40 were female and 81 were male, with a median age of 40 years. All samples were from India. The features of the patients included in the study are summarized in Table 3.

We found 79 negative and 42 positive samples, which comprise all 42 samples of the failure stage of CML disease. In 42 positive samples, a total of 63 variants were detected, with varying allele frequencies ranging from 2.3% to 93.41% (refer to Table 4). Nine samples were detected to have compound mutations, whereas the rest of the samples were found to have single mutations. The analysis of the variants with respect to the sensitivity to the available first, second, and third generations of TKIs is determined and summarized along with the results.

**Table 4:**
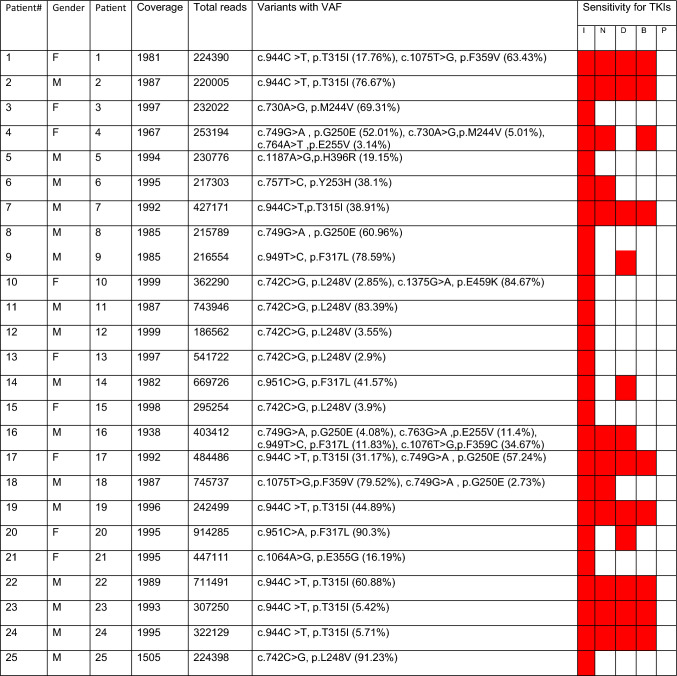

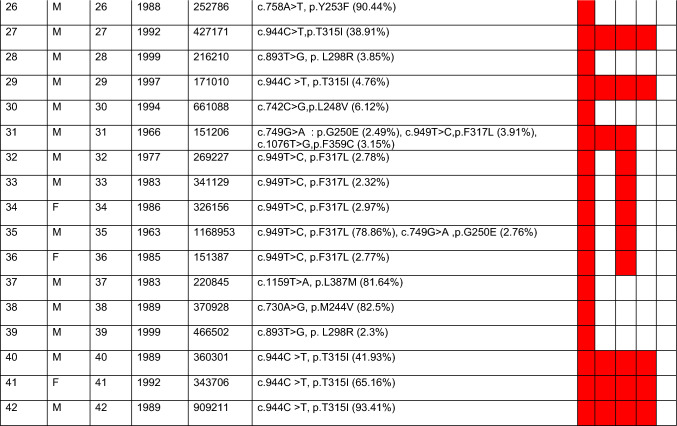
Data of all the samples considered for the KD mutation detection and variants detected with their respective allele frequency and their sensitivity for TKIs.

Gender column, M = Male, F = Female.

VAF = Variant allele frequency.

*TKIs full form: I–Imatinib, N–Nilotinib, D–Dasatinib, B–Bosutinib, P–Ponatinib.

Coverage and VAF provided here, is calculated by Ion Reporter software. Cells highlighted in red indicate poor sensitivity towards the specific TKI in the respective column.

## Discussion

The National Comprehensive Cancer Network (NCCN) and the European Leukemia Net (ELN) both have recommended *BCR::ABL1* KD mutation testing in CML patients who do not achieve an optimal response to TKI therapy^16,31,32^. ELN in 2011 recommended SS as the gold standard for *BCR::ABL1* KD mutation screening, with limited sensitivity (15–20%) at that time, while NCCN has no guidelines for the same^31^. In the recent past, NGS and droplet digital PCR (ddPCR) have emerged as more sensitive techniques to detect minor subclones. However, both approaches present certain limitations. Polivkova et al.^33^ have reported the feasibility of KD mutation detection in gDNA with 0.1% sensitivity by ddPCR (droplet digital PCR). In the ddPCR method, mutation-specific primer probe development and multiplexing is a challenge to cater to all the mutations, so it is limited to 10–15 mutations per experiment. At present, many laboratories are in the process of implementing NGS technology and integrating NGS results into the diagnostic algorithms of patients with various hematological malignancies.

NGS plays an important role in mutation detection at very high sensitivity, which plays a pivotal role in therapy selection in the case of CML disease treatment. Many studies have already established that NGS can detect the presence of mutations 12–15 months before they are identifiable by SS^22^. Traditional methods available can detect mutations, but many of them are not easily accessible, and others have a lesser sensitivity. Commonly available and highly used method for KD mutation analysis is sanger sequencing method which detects the mutations at the 10–20% sensitivity. The sensitivity is the lowest among all available methods, but its cost effectiveness, faster turnaround time, and ability to detect novel mutations within a targeted region make it a workable choice. Many other methods that have been reported till now with varying limits of detection and sensitivity are summarized in Table 5. Amongst all the methods reported, NGS is easy to adapt and also has very good sensitivity to be used as a routine clinical assay.

**Table 5:** Comparison of various methods used till date for the detection of KD mutations, pros and cons of each method, and their sensitivity.

SN	Method	Sensitivity	Pros	Cons	References
1	Sanger Sequencing (Direct Sequencing)	15–25%	Mutation characterization, high confidence, Accurate, Fast, Cost effective, Novel Mutation detection	Less sensitive	^34,35^
2	Sanger sequencing (In-direct Sequencing, cloning & sub cloning)	15–25%	Mutation characterization, high confidence, Accurate, Fast, Cost effective, Novel Mutation detection	Less sensitive, very tedious and time consuming	^12,36,37^
3	Pyrosequencing	5%	High sensitivity and specificity	Always needs internal positive and negative controls, tedious	^12,36,37^
4	Denaturing high-performance liquid chromatography- DHPLC (WAVE)	1%	Cost effective and high throughput	Wild type DNA spiking required, complex processing makes it difficult and error prone , cannot characterize novel mutation within targeted region	^31,37–41^
5	High resolution melt curve analysis (HRM)	5–10%	Cost effective and high throughput	Cannot characterize novel mutation within targeted region, effective on small amplicon size needs many amplicons to target complete region	^42,43^
6	Amplification refractory mutation system polymerase chain reaction (ASO-PCR)	0.01–0.001%	High sensitivity and specificity, Fast to perform	Specificity decreases in case of mutations in close proximity, Cannot detect novel mutation within targeted region, Very tedious if performed for all the mutations together	^37,42–47^
7	Ligation polymerase chain reaction (L-PCR)	0.1–0.05%	High sensitivity and specificity, Fast to perform	Multiple probes required for targeting all the mutations, Cannot detect novel mutation within targeted region	^48^

In our knowledge, there is no commercial kit or assay with either European Conformity (CE)-marked for in vitro diagnosis (CE-IVD) or Food and Drug Administration (FDA)-approved commercial kits available for NGS-based *BCR::ABL1* KD mutation. Though there are many good myeloid panels available on the market from Thermofisher, Qiagen, Illumina, Archer, etc., they are not enriching the transcript P210 or P190 before sequencing. A recent study that used a DNA-based method to detect kinase domain mutations found that the method had a 92% sensitivity and an 81.6% specificity^49^. The use of DNA as a starting material may have contributed to a decrease in the assay's specificity. The study employed the same methodology as current myeloid panels, using nine primers to amplify exons 4 through 10 of the KD from DNA. The guideline states that fusion transcripts cannot be screened for mutations using DNA as a starting material because resistant mutations occur in the fusion transcript rather than in DNA, diluting the mutation fraction^4,50^. Assays that directly amplify the KD region from the *ABL* gene can give false amplification, but a nested PCR-based approach corrects this pitfall as it amplifies only when *BCR::ABL1* fusion copies are present in the given sample. Ultimately, mutation screening by this direct approach would mainly have untranslocated *ABL1* as a substrate, which ultimately dilutes the mutations down to a level that might be undetectable even by NGS^4^. The protocol presented in this study is very fast, accurate, reproducible, and easy to implement for any lab that routinely uses any of the assays on the IonTorrent platform. The derived sensitivity of the assay is 2%, which is well within the defined range. 1–3% for NGS-based assays^4^. Despite the higher sensitivity and specificity of the current NGS-based assay in detecting variants, the chances of obtaining false positive or false negative results cannot be eliminated due to various factors. As this assay uses nested PCR amplification of fusion transcript followed by NGS, cases with early loss of mismatch repair (deep molecular remission) can be determined as false negatives due to lower target transcript abundance. However, it is worth noting that detection in such cases can only be possible by target-specific ddPCR methods, which is difficult until the variant is known. Factors for the false-positive results can be PCR artifacts, sequencing errors, base-call accuracy, etc. In order to improve the accuracy of the sequencing technique, unique molecular identifiers can be a good option instead of normal barcodes. To avoid any false calls, visualization of variants with IGV is strongly recommended.

In this study, we have successfully demonstrated the protocol for kinase domain mutation analysis by the NGS method using the IonTorrent Gene Studio S5 sequencing platform. This method uses an RNA-based approach to detect mutations through deep sequencing. As per our review of the literature, there are few studies available that have reported protocols for kinase domain mutation analysis by the NGS method^18–22,24,27,51,52^, and many of them are based on Illumina platforms, one of which is the Roche platform, which is now obsolete. Amplification-based approaches are considered better than probe hybridization and capture-based approaches due to the lesser requirement of template material, simpler sample preparation, and lower time consumption. However, in the case of CML samples, starting material is not an issue, but the accessibility of ion torrent platforms in the majority of clinical laboratories makes the amplification-based method a preferred choice. The use of shorter lengths (200–250 bp) and overlapping amplicon strategies provides improved coverage uniformity in GC-rich regions, which is also considered a drawback of amplicon sequencing^53^. Moreover, Ion torrent platforms are accessed by many diagnostics labs globally due to their targeted panels and ease of bioinformatics analysis as they use user-friendly ion reporter interface. A comparison table (Table 6) has been provided in which details of similar assays developed in the recent past, their methodology and sensitivity, as well as sample size, are mentioned. This is the first study from India that represents the kinase domain mutation analysis data of more than 160 samples using a nested PCR approach for targeting *ABL* transcript region.

**Table 6:** Comparison of studies of kinase domain mutation analysis by next-generation sequencing methods.

SN	Study size	Methodology used	Assay sensitivity	Year of publication	Country	Remarks	Reference
1	15	RNA + P210 specific amplification + Nested PCR	1%	2014	Czech Republic	GS Junior instrument (Roche Diagnostics)	^22^
2	121	RNA + P210 specific amplification + Fragmentation based Library Prep	1%	2019	UK	Miseq (illumina) + No nested PCR	^24^
3	33	RNA + P210 specific amplification + Nested PCR	1%	2013	Italy, Czech Republic, Germany	GS Junior instrument (Roche Diagnostics)	^27^
4	79	RNA + P210 specific amplification + Nested PCR	1%	2016	Italy, Czech Republic	GS Junior instrument (Roche Diagnostics)	^18^
5	51	RNA + P210 specific amplification + Nested PCR	1%	2016	Italy, Czech Republic	GS Junior instrument (Roche Diagnostics)	^54^
6	47	RNA + P210 specific amplification + Nested PCR	< 5%	2018	Turkey	GS Junior instrument (Roche Diagnostics)	^52^
7	508	RNA + P210 specific amplification + Fragmentation based Library Prep	Not Available	2016	USA	Ion Torrent Personal Genome Machine	^21^
8	162	DNA + P210 specific amplification + Fragmentation based Library Prep	1.0E − 4	2022	Spain	Ion GeneStudio S5	^49^
9	31	DNA + P210 specific amplification + Fragmentation based Library Prep	3%	2020	Czech Republic	Illumina NGS Platform	^55^
10	36	RNA + P210 specific amplification + Fragmentation based Library Prep	Not Available	2022	India	Illumina NGS Platform	^55^
11	97	RNA + P210 specific amplification + Hybrid Capture based Library Prep	3%	2021	India	Illumina NGS Platform	^55^

### Assay performance and findings

**Figure 6:**
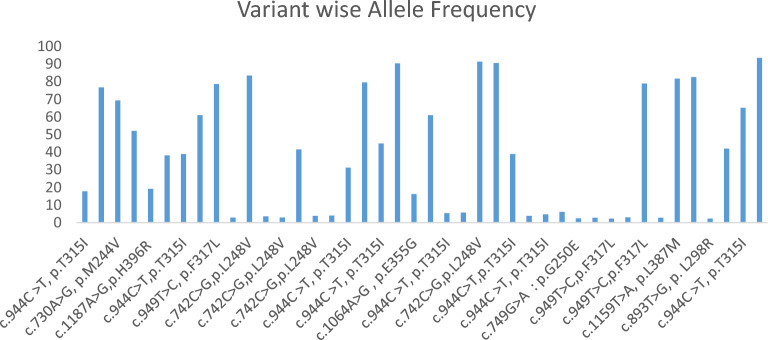
Variant-wise allele frequency in all 63 mutations in 42 positive samples ranges from 2.32 to 93.41%.

**Figure 7:**
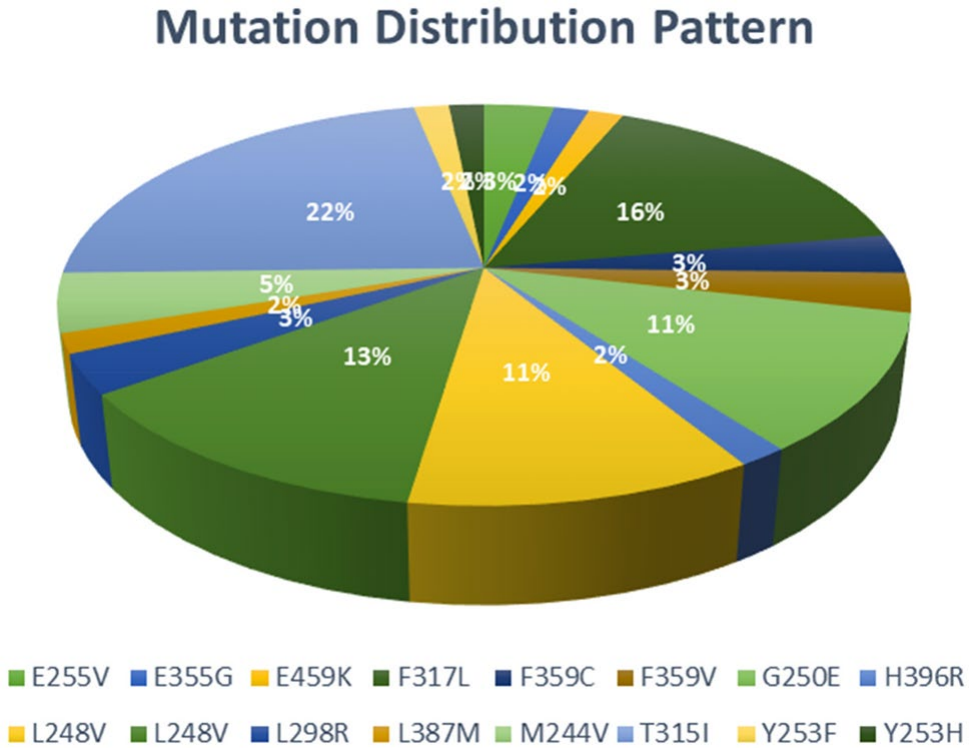
Distribution of total 63 mutations detected in 42 positive samples and their percentage.

We found mutations in 34.71% of CML patients, whereas 66.94% of CML patients were found to be negative. In all the positive samples (n = 42), variant T315I was found most frequently (n = 14) at 22.22%, followed by F317L (n = 10) at 15.87%, L248V (n = 8) at 12.70%, and the least detected mutations were E355G (n = 1), E459K (n = 1), H396R (n = 1), L387M (n = 1), Y253F (n = 1), and Y253H (n = 1). The allele frequency of each detected mutation is represented by bar charts (Fig. 6). In our study, we observed that 8 samples out of 42 samples with mutations were found to have compound mutations. The mutational distribution pattern and frequency of 63 mutations detected in positive samples (n = 42) is represented by the pie chart in Fig. 7.

In all of these eight samples, one mutation was found at > 20% frequency, and the rest were between 2 and 20% VAF. This infers that if such samples were being sequenced by SS, one primary mutation with good VAF would have been captured, missing out on others. Variants detected in each positive sample and their inference with respect to sensitivity for TKIs are provided in the result section (Table 4). Sample 1 had two variants, T315I and F359V, at 17.76 and 63.43% VAF, respectively. If such samples were detected by SS, it would have been inferred as having poor sensitivity for Imatinib and Nilotinib due to the lower sensitivity of the SS method, but in reality, this patient is also poorly sensitive for Dasatinib and Bosutinib as they also contain the T315I variant in the same sample. Such an example proves the importance of using sensitive methods like NGS for such assays. In patient 4, the primary variant detected with a high allele frequency was G250E (52.01%), along with other variants like M244V (5.01%) and E255V (3.14%). In this case, the E255V variant is detected at low concentration, which means it’s just started developing and will be visible after some months or years, depending on the progress of the disease. Identification of such variants will help in making the decision to skip the second generation of TKIs instead of waiting for patients to become resistant and move directly to the third generation of TKI. In sample 10, despite having two variants (L248V and E459K) detected, there is not much difference in the decision to select TKI as both variants confer resistance to Imatinib only. Sample 16, considered a failure as per ELN guidelines by the *BCR::ABL* quantitative assay, was found to have four compound variants, which is the highest in this study. This sample was detected to have F359C at 34.67%, followed by F317L at 11.83%, E255V at 11.4%, and G250E at 4.08% VAF, making it poorly sensitive to TKIs like Imatinib, Nilotinib, and Dasatinib. For confirmation, this sample was repeated twice, and all four variants were present in the sample in both attempts. Sample 17 was found to have two variants, T315I with a 31.17% allele frequency and G250E with a 57.24% allele frequency, which is also interestingly detectable by SS.

For the simplification of the analysis part, we used Thermofisher ion reporter software at the backend by applying customized bed files. This bed file contains a targeted region of the *ABL* gene with all the hotspot variants marked as per Soverini et al.^4^ allowing ease of analysis without the need for a skilled bioinformatician. Since our pipeline uses all the databases at the backend, analyses of the entire region of interest are covered automatically and systematically. As per the validation assay performed, we have considered the LOD for this assay as 2% and the lowest read count per sample as 100,000 reads. This assay covers all aspects of NGS made with the hotspot variant. More importantly, by this assay, the IGV analysis of each and every variant can be checked for the true variant. In the recent past, one study has reported having validated similar assays using a DNA-based approach that claimed to have similar sensitivity to RNA-based assays, but variants identified from the DNA or fusion will be a challenging issue.

### Assay unique features

In summary, our newly developed assay can be used for kinase domain mutation analysis from clinical samples with a very good sensitivity of 2%, which is in the well-acceptable range of 1–3%, and is available on the commonly used IonTorrent platform. The same assay can be used for R/R Ph-positive ALL patients as well, as it contains primers for the amplification of the ALL-specific transcript (P190) but needs to be validated. This ability of the assay to detect low-level variants and even compound variants makes it very important for the selection of appropriate TKIs for CML patients. This method can be easily adopted in clinical practice to detect the KD mutation status in CML patients with failure or warning status and is very useful for TKI selection.

### Supplementary Information

Supplementary Information.

## Data Availability

The datasets generated during and/or analysed during the current study were uploaded to NCBI with accession ID: PRJNA1125133 and can be found at the following link: https://www.ncbi.nlm.nih.gov/sra/SRX24995656[accn].
