# Supplementary material for: Validation of a novel NGS based BCR::ABL1 kinase domain mutation detection assay in Indian cohort
Source: Sci Rep. 2024 Jul 8;14:15745. doi: 10.1038/s41598-024-66310-8 (PMC11231265; doi:10.1038/s41598-024-66310-8)
Supplement: Supplementary file 1 — Supplementary Information. [file 41598_2024_66310_MOESM1_ESM.docx]

**Supplementary Material**

Validation of a novel NGS based BCR-ABL1 kinase domain mutation detection assay in Indian cohort

**Protocol**

**Total RNA extraction**

Total RNA was extracted from EDTA blood or paxgene tubes using the QIAamp RNA Blood Mini Kit (Cat no. 52304) as per the manufacturer's instructions. RNA quality was analyzed using gel electrophoresis to check the integrity of the RNA, and quantification was done using a Qubit 4.0 fluorometer by the Qubit RNA HS assay (Cat no. Q10210).

**cDNA preparation and fusion specific amplicon generation**

500 ng to one microgram of total RNA was converted to cDNA in a final volume of 10μL using the NGS reverse transcription kit (Cat no. A45003) according to the manufacturer’s instructions.

A first step of amplification aimed to co-amplify the BCR::ABL1 fusion transcript breakpoint and the kinase domain (KD), thus excluding the untranslocated ABL allele from the analysis. This was performed by polymerase chain reaction (PCR) with a forward primer located either on BCR exon 1a (5’‐CAACAGTCCTTCGACAGCAG‐3’; for patients known to have the e1a2 BCR‐ABL fusion, corresponding to the p190 BCR‐ABL1 oncoprotein) or on BCR exons 12‐13 border (5’‐GAGCAGCAGAAGAAGTGTTTCAGA‐3’; for patients known to have either the b2a2 or b3a2 BCR‐ABL fusion, corresponding to the p210 BCR‐ABL1 oncoprotein). The reverse primer was located on ABL, exon 10 (5’‐CTTGGAGTGAGGCATCTCAG‐3’). Amplification was performed using Platinum™ SuperFi II PCR Master Mix (Cat no. 12368010). Reactions were carried out in a 20μL final volume containing 2.5 μL cDNA, 10μL Platinum™ SuperFi II PCR Master Mix, and 1.25μL of 10 pM of each primer (forward and reverse). An initial denaturation step of 5 min at 95 °C was followed by amplification for 30 cycles (denaturation for 30’’ at 95 °C, annealing for 30’’ at 60 °C, extension for 2’30’’ at 72 °C), and a final extension for 7 min at 72 °C. The resulting amplicon of P190 or P210 was purified using 0.8X Alexgen DNA Magbeads-NGS (Cat no. 1013) to remove dimers and PCR components used in the reaction. The final amplicon was quantified using a Qubit florometer (version 4.0), and 10 ng of the same will be used for the next step of library preparation.

Purified amplicon was subjected to amplification using kinase domain region-specific primers, which were divided into two separate pools to prevent adjacent amplicon generation and to increase the specificity of the primers. Using these primer pools, target amplification was done using Ion AmpliSeq™ Library Kit 2.0 (Cat no. 4480442), and the rest of the protocol was followed as per the manufacturer's instructions. Ampure beads mentioned in the protocol were replaced by Alexgen DNA magbeads in all the steps. The final library was quantified using the Ion Library TaqMan™ Quantitation Kit (Cat no. 4468802) to determine the concentration of the library. Based on the quantification results, dilution was carried out, and 100 pM of the final library was sequenced using the Ion 540™ Kit-Chef (Cat no. A30011) and the Ion 540™ Chip Kit (Cat no. A27766).

**Data Analysis**

Bed files containing targeted regions of the transcript NM_005157.6 with annotation of kinase domain-resistant hotspot mutations were prepared using genome build Hg19 and uploaded to the ion reporter software. Generated data was subjected to analysis by using generated bed files as references, and variants were called by the software with annotations.

**Sensitivity Determination Experiment**

For sensitivity determination, one positive sample was selected with variant F317L at 11.83% VAF, which was serially diluted in a 1:1 ratio with the RNA sample of a known negative patient for further three times (Table 2 in supplementary data). Diluted samples were subjected to cDNA preparation, transcript enrichment, library preparation, data generation, and analysis as mentioned in the methodology segment.

**Reproducibility Experiment**

A total of five patient samples were used for reproducibility experiments, which included three positive and two negative samples. All the samples were processed as total RNA for data generation as per the methodology mentioned and subsequent analysis.

Table 1: Determination of LOD by using a CML sample positive for c.949T>C: p. Phe317Leu variant with 11.83% VAF.

| **Sample** | **Run** | **Expected VAF (%)** | **Detected VAF (%)** | **Coverage** |
| --- | --- | --- | --- | --- |
| Undiluted | Run#1 | 11.83 | 11.83 | 1967 |
| 50% diluted | Run#2 | 5.92 | 3.91 | 1971 |
| 75% diluted | Run#2 | 2.96 | 2.78 | 1977 |
| 87.5% diluted | Run#2 | 1.48 | 2.32 | 1983 |

VAF – Variant allele frequency, LOD – Limit of detection

Table 2: Reproducibility results of CML sample positive and negative samples with variant details, VAF, Coverage

| Patients and Replicates | Variant Detected | VAF | Coverage |
| --- | --- | --- | --- |
| Patinet#1 + Replicate#1 | ABL1: c.949T>C : p.Phe317Leu | 78.86% | 1938 |
| Patinet#1 + Replicate#2 | ABL1: c.949T>C : p.Phe317Leu | 80.25% | 1940 |
| Patinet#1 + Replicate#3 | ABL1: c.949T>C : p.Phe317Leu | 84.77% | 1925 |
| Patinet#2 + Replicate#1 | ABL1 : c.944C>T : p.Thr315Ile | 4.76% | 1997 |
| Patinet#2 + Replicate#2 | ABL1 : c.944C>T : p.Thr315Ile | 2.31% | 1987 |
| Patinet#3 + Replicate#1 | ABL1: c.749G>A : p.Gly250Glu | 60.77% | 1960 |
| Patinet#3 + Replicate#2 | ABL1: c.749G>A : p.Gly250Glu | 56.14% | 1961 |
| Patinet#3 + Replicate#3 | ABL1: c.749G>A : p.Gly250Glu | 60.96% | 1911 |
| Patinet#3 + Replicate#4 | ABL1 : c.749G>A : p.Gly250Glu | 68.41% | 1966 |
| Patinet#4 + Replicate#1 | Negative | NA | NA |
| Patinet#4 + Replicate#2 | Negative | NA | NA |
| Patinet#4 + Replicate#3 | Negative | NA | NA |
| Patinet#5 + Replicate#1 | Negative | NA | NA |
| Patinet#5 + Replicate#2 | Negative | NA | NA |

VAF – Variant allele frequency

Table 3: Validation data of known positive and known negative samples by SS and newly developed NGS assay.

| **Samples** | **SS Method** | **Variants with allele frequencies detected by NGS** |
| --- | --- | --- |
| 1 | T315I | T315I-27.76% & F359V-63.43% |
| 2 | T315I | T315I-76.67% |
| 3 | M244V | M244V-69.31% |
| 4 | G250E | M244V - 5.01%, G250E-52.01%, Q255V-3.14%, F359I-16.80%, F359C-2.90% |
| 5 | H396R | H396R- 19.15% |
| 6 | Y253H | Y253H-38.10% |
| 7 | T315I | T315I- 38.91% |
| 8 | Negative | L298R-3.85% |
| Rest 13 Negative samples were also detected negative by NGS | | |

SS Method- Sanger sequencing method
